# A mobile technology intervention to reduce sedentary behaviour in 2- to 4-year-old children (Mini Movers): study protocol for a randomised controlled trial

**DOI:** 10.1186/s13063-017-1841-7

**Published:** 2017-03-03

**Authors:** Katherine L. Downing, Jo Salmon, Trina Hinkley, Jill A. Hnatiuk, Kylie D. Hesketh

**Affiliations:** 1 Deakin University, Institute for Physical Activity and Nutrition (IPAN), School of Exercise and Nutrition Sciences, Geelong, VIC Australia; 2 School of Science and Health, Western Sydney University, Penrith, NSW 2751 Australia

**Keywords:** Sedentary behaviour, Screen time, Television viewing, Sitting time, Early childhood, Randomised controlled trial, mHealth, Text messaging, SMS

## Abstract

**Background:**

Sedentary behaviour (e.g. television viewing, sitting time) tracks over time and is associated with adverse health and developmental outcomes across the lifespan. Young children (5 years or younger) spend up to 12 h/day sedentary, of which around 2 h is spent in screen time (e.g. watching television). Interventions to reduce sedentary behaviour in early childhood report mixed results and many have limited potential for scalability. Mobile phones offer a wide-reaching, low-cost avenue for the delivery of health behaviour programmes to parents but their potential to reduce young children’s sedentary behaviour has not been widely tested. This study aims to test the feasibility and efficacy of a parent-focused, predominantly mobile telephone-delivered intervention to support parents to minimise the amount of time their child spends using screens and in overall sitting time.

**Methods/design:**

Mini Movers is a pilot randomised controlled trial recruiting 100 parents and children. Inclusion criteria include having a child aged between 2 and 4 years, being able to speak, read and write English, and smartphone ownership. Participants will be randomised to the intervention or a wait-list control group at a 1:1 ratio. Intervention group parents will receive printed materials including a content booklet and goal-checking magnet and will participate in a one-on-one discussion with the interventionist to plan two goals to reduce their child’s sedentary behaviour. Subsequently, the intervention will be delivered over 6 weeks via personalised and interactive text messages promoting positive health behaviours (strategies for decreasing screen time and overall sitting time), goal setting and self-monitoring. Outcomes to be assessed include intervention feasibility and children’s screen time and objectively-assessed sitting time.

**Discussion:**

Few studies have used mobile phone technology to deliver health behaviour programmes to parents of young children. Findings will inform the development of larger-scale interventions to reduce sedentary behaviour during early childhood.

**Trial registration:**

Australian New Zealand Clinical Trials registry, identifier: ACTRN12616000628448. Prospectively registered on 16 May 2016.

**Electronic supplementary material:**

The online version of this article (doi:10.1186/s13063-017-1841-7) contains supplementary material, which is available to authorized users.

## Background

High levels of sedentary behaviour have been associated with adverse health and developmental outcomes across the lifespan [1–4]. Some sedentary behaviours, such as television viewing, have been shown to track over time [5, 6], with early childhood (i.e. birth through 5 years) being recognised as a critical period in which sedentary behaviour habits are established [7]. Guidelines for sedentary behaviour in Australia and internationally recommend that children aged 2 to 5 years should have less than 1 h per day of screen time [8, 9]. Additionally, it is recommended that situations that restrict movement, i.e. in a car seat, stroller or high chair, should be minimised for children aged 5 years and younger [8–10]. Research has shown that 2- to 5-year-old children are spending on average 2 h per day in screen time [11–14], with only around a quarter of these children meeting current recommendations of 1 h or less per day [11, 12, 15]. Children of this age are also spending up to 12 h per day in any form of sedentary behaviour when assessed objectively [16], and approximately 2 h per day in situations that restrict movement [17]. This suggests that there is considerable scope to reduce sedentary behaviour in young children. Feasible, acceptable and effective interventions are required during the early childhood period, prior to the establishment of less than optimal levels of sedentary behaviour.

A recent review of interventions to reduce screen time in children younger than 12 years identified 47 studies, of which only 13 targeted children under the age of 6 years [18]. All of the studies targeting young children were conducted in the United States and the majority (11 studies) were delivered in either preschools or clinic- and Women, Infant and Children (WIC)-based (federally assisted programs for low-income mothers and children in the United States) settings, with the remaining two conducted in the home [18]. Schmidt et al. noted that the largest reductions in television viewing across all studies (i.e. all age groups) were seen in home-based settings, and suggested that high levels of parental involvement are important for intervention effectiveness [18]. An earlier review of obesity-prevention interventions during early childhood similarly suggested that the lack of parental involvement in preschool interventions may explain the lack of significant results [19]. Findings from Schmidt et al. [18] highlight the relative paucity of interventions in early childhood, and also the need for interventions that are scalable and have large reach.

Given the rapid and wide adoption of mobile phone usage across most adult age and demographic groups [20], health behaviour programmes are increasingly being delivered by mobile phone technology [21]. In particular, text messages, or short message services (SMS), are considered to be a wide-reaching, low-cost channel for the delivery of health behaviour programs [22]. Text messages are also instantaneous and convenient, in that individuals can read them in their own time. Moreover, they can be individually tailored, which has been shown to have positive effects on behaviour change and reduces attrition [22]. However, to date, text message interventions have largely focused on preventative health behaviours in adults, such as smoking cessation, and clinical care [22]. Few studies have used text messages in programs targeting child and adolescent health behaviours [23]. However, a recent pilot intervention delivered largely via text messages to parents, that focused on healthy lifestyle behaviours for overweight and obese preschoolers, showed significant improvements in parental knowledge around nutrition and physical activity [24]. Moreover, the intervention was found to be both feasible and acceptable for parents of young children [24] suggesting such delivery modes hold promise in this population group. Thus, the aim of this study is to test the feasibility and efficacy of a parent-focused, predominantly mobile telephone-delivered intervention to support parents to minimise the amount of time that their 2–4-year-old children spend in sedentary behaviour.

## Methods/design

### Overview

**Fig. 1:**
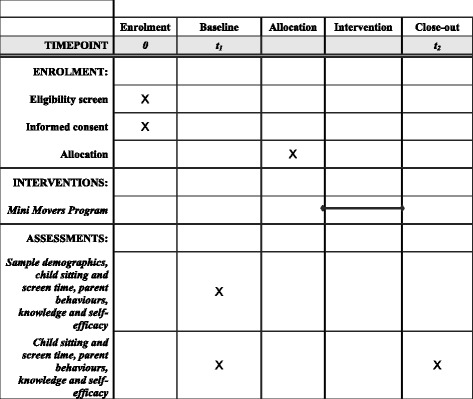
Schedule of enrolment, interventions and assessments

This protocol describes a two-armed, pilot randomised controlled trial (RCT) to evaluate the feasibility and efficacy of a parent-focused, predominantly mobile phone-delivered intervention to reduce sedentary behaviour in 2- to 4-year-old children. The protocol is guided by the Standard Protocol Items: Recommendations for Interventional Trials (SPIRIT) statement [25] and the Consolidated Standards of Research Trials (CONSORT) – EHEALTH guidelines [26, 27]; Additional file 1: shows the completed SPIRIT Checklist (see Additional file 1). Figure 1 provides an overview of the schedule for enrolment, interventions and assessments [25].

### Recruitment

Participants will be recruited in Melbourne, Australia through existing playgroups, parent-focused websites and social media, and snowball sampling.

#### Playgroups

In Australia, playgroups are informal gatherings for parents, caregivers and their children prior to the commencement of primary school [28]. In addition to providing opportunities for children to interact, play and develop, playgroups also provide a supportive environment for parents to share experiences about parenting [28]. Hence, they may provide an ideal setting for recruiting parents for child behaviour interventions, as parents may be more receptive in a setting where other child or parenting issues are usually discussed. The Playgroup Victoria public website (http://www.playgroup.org.au) provides names and contact details for the lead parents and/or playgroup leaders of playgroups across the state. Playgroups within a 10-km radius from the study site (Deakin University, Burwood Campus, Melbourne, Australia) will be identified via the website and randomly selected. Lead parents/playgroup leaders will be contacted by email and/or phone initially to gauge interest in the intervention programme and determine if the families attending the playgroup meet the inclusion criteria for the study. If the leader expresses interest and families appear to be eligible, a researcher will visit the playgroup to explain the study to the parents and provide them with plain language statements, Consent Forms and contact details of the research team. Parents will be able to provide written consent on the day of the recruitment visit, or will be able to return their consent form by email, post or in person at the baseline visit the following week. Alternatively, for more structured playgroups where a recruitment visit may not be possible, flyers with brief programme information will be delivered for playgroup leaders to hand out to parents. Interested parents will then be able to contact the research team directly for more information.

#### Websites and social media

Individuals and organisations that provide services to, or work, volunteer or collaborate with, the target population (i.e. parents with young children; e.g. reputable parenting blogs) will be contacted and asked to post information about the study on their website, community groups, blog or social media (e.g. Twitter, Facebook). Information on websites and social media will be the same as that included on the flyer and will instruct parents who are interested in participating to contact the research team directly for more information.

#### Snowball sampling

Parents participating in the programme will be asked to pass on the details of the study and research team to any friends that may be interested in participating. Interested parents will be able to contact the research team directly for more information.

### Inclusion criteria

Parents will be eligible to participate if they have a child aged 2 through 4 years, are able to freely give informed consent, can speak, read and write fluent English and own a mobile phone.

### Sample size

As this is a pilot study, a sample of 100 participants will be recruited. This sample size will provide feasibility data for the critical recruitment and compliance parameters and also for the estimation of the standard deviation of sitting time and screen time (both continuous variables) [29].

### Randomisation

Participants will be randomised to the intervention or wait-list control at a 1:1 ratio after baseline data collection. If more than one parent is recruited in a playgroup, randomisation will occur at the group level to avoid potential contamination. A computer-generated random number schedule will be developed by a researcher (not part of the research team) who has no contact with the participants. Allocation will be concealed by sealed, opaque envelopes, which will be opened and revealed to the researcher and participant(s) after baseline data collection to minimise selection and measurement bias.

### Mini Movers intervention

#### Intervention content

The intervention content for Mini Movers was developed based on evidence-based guidelines for sedentary behaviour and active play in early childhood [8], and guided by the CALO-RE taxonomy of behaviour change techniques [30] and Social Cognitive Theory [31]. The intervention comprises a content booklet, a one-on-one goal-setting discussion with the interventionist, and regular, personalised text messages for a period of 6 weeks. Intervention strategies focus on increasing parental knowledge, building self-efficacy, setting goals and providing reinforcement. Table 1 presents intervention strategies mapped to theoretical constructs.

**Table 1:** Intervention strategies mapped to theoretical constructs

Strategies	Theoretical constructs
Provide parents with evidence-based guidelines for sedentary behaviour	SCT: Knowledge
Provide parents with ideas for minimising sedentary behaviour (e.g. changing activities such as drawing or painting from sitting down to standing up, setting screen time rules, removing screens from bedrooms, leading by example)	SCT: Self-efficacy CALO-RE: Provide instruction on how to perform the behaviour CALO-RE: Environmental restructuring CALO-RE: Prompt identification as role model/position advocate
Provide parents with alternatives to sedentary behaviour (e.g. new activities to try, providing practical ideas for entertaining children when cooking dinner)	SCT: Knowledge SCT: Self-efficacy SCT: Access CALO-RE: Provide information on *where and when* to perform the behaviour CALO-RE: Provide instruction on how to perform the behaviour
Assist parents to set goals to reduce screen time and overall sitting time (e.g. to limit their child’s screen time to 30 min per day)	SCT: Goal setting CALO-RE: Goal setting (behaviour)
Educate parents about benefits of reducing sedentary behaviour and increasing active play (e.g. detrimental effects of screen time on sleep, benefits of active play on development)	SCT: Knowledge CALO-RE: Provide information on consequences of behaviour in *general*
Provide parents with a goal-checking magnet to monitor their progress with their goals	CALO-RE: Prompt self-monitoring of behaviour
Send weekly goal-check SMS	CALO-RE: Prompt review of behavioural goals
Provide parents with positive reinforcement and suggest rewards (e.g. an afternoon in the park with their child) when goals are met	CALO-RE: Prompt rewards contingent on effort or progress towards behaviour SCT: Reinforcement

*SCT* social cognitive theory, *SMS* short message service

##### Intervention materials

After baseline measures have been completed and randomisation has taken place, participants in the intervention group will receive their intervention materials, including a content booklet, goal-checking magnet and a Move and Play Every Day: National Physical Activity Recommendations for Child 0–5 Years brochure [8]. The content booklet provides an overview of the Mini Movers programme and text messages that parents will receive, suggests ideas for reducing sedentary behaviour and increasing active play, and introduces goal setting. At this time, participants will have a one-on-one discussion with the interventionist to set their goals. Participants will be asked to set two goals around their child’s sedentary behaviour; specifically, one screen time goal (e.g. to limit their child’s screen time to 60 min per day) and one overall sedentary behaviour goal (e.g. to walk to local destinations without the pram on 3 days per week). The interventionist will assist participants in identifying and setting SMART (Specific, Measurable, Attainable, Relevant and Time-bound) goals. The goal-checking magnet provided to participants was designed to help track their progress with their two goals for the duration of the programme (6 weeks).

##### Text messages

Personalised, interactive text messages will be the main mode of delivery for the intervention. Participants will receive four text messages per week for 6 weeks (24 texts in total). The text messages will include ideas for limiting and displacing their child’s screen and sitting time, active play ideas, and monitoring and encouraging achievement of individual goals. Some text messages will include links to reputable websites for further information.

The text messages will be tailored to the participant’s name, child’s name, behaviour goals and the interventionist’s name, as evidence suggests that personalisation of text message programs encourages behaviour change and reduces attrition [22]. Text messages will be sent on specific dates at specific times. Participants will be asked to nominate a preferred time of day to receive messages (e.g. early morning, late afternoon); however, some text messages are designed to be delivered at specific times of the day to coincide with specific activities (e.g. ideas for keeping their child entertained without screens whilst cooking dinner). Examples of the text messages include: “Hi «parent». We know that entertaining «child» can be difficult sometimes without using the TV or other screens. Check out this picture for some ideas! «link». Mini Movers”; and “«Parent», get «child» to help make some playdough! Here’s a great recipe with no cooking required: «link». Remember, encourage «child» to stand up while playing with it! Mini Movers”. Two-way texting will be used for the goal monitoring. This will require participants to respond to the message enquiring as to whether they met their goal, to which the researchers will reply with a predefined response, depending on whether the goals were achieved or not.

### Wait-list control

Participants randomised to the wait-list control group will receive the full intervention (i.e. goal-setting discussion, content booklet, goal-checking magnet and text messages) after post-intervention assessments have been completed.

### Measures

**Fig. 2:**
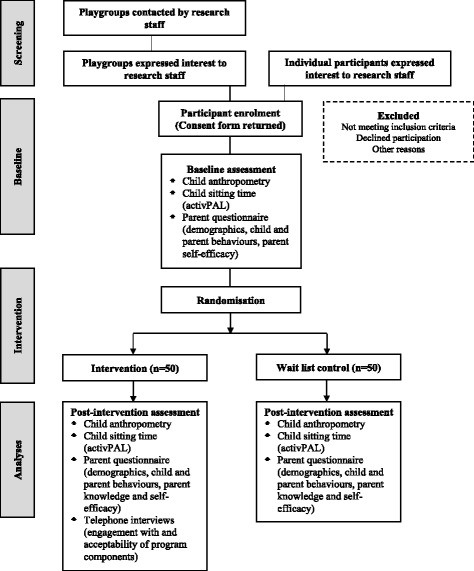
Trial flow diagram

The primary outcome of this trial is feasibility, which will be measured with programme metrics, recruitment, and participant self-reported data post-intervention. The secondary outcomes are children’s objectively measured sitting time and parent-reported screen time, and parent behaviours, knowledge and self-efficacy for limiting their child’s sedentary behaviour assessed pre and post intervention (Fig. 2). Children’s sitting time will be measured objectively using *activ*PAL™ accelerometers worn pre and post intervention. All other secondary outcomes, potential mediators and demographics (apart from the child’s Body Mass Index (BMI)) will be parental proxy-reported using an online survey delivered by Qualtrics (Qualtrics Labs, Provo, UT, USA), completed pre and post intervention.

#### Primary outcome

Feasibility will be measured by recruitment numbers, programme metrics and self-reported participant data, as described below. The process evaluation is informed by the Process-Evaluation Plan for Assessing Health Programme Implementation [32] and the e-CONSORT guidelines [27].

1. *Recruitment and retention.* Recruitment will be measured by: the proportion of playgroups interested in the study (i.e. the proportion of playgroups allowing a visit by the research team or distribution of flyers); the proportion of eligible parents within playgroups consenting; the number of parents recruited via social media and snowball sampling; and the time taken to recruit the sample. Retention will be measured by the proportion of participants providing measures at the end of the study
2. *Intervention delivery and fidelity.* Intervention delivery and fidelity, i.e. successful delivery to protocol, will be measured by system reports (e.g. delivered text messages), reports of technological difficulties (e.g. parent self-report of mobile phone downtimes, lack of Internet access) and auditing of protocol compliance in delivery of one-on-one goal-setting discussions by a single researcher
3. *Dose delivered and engagement in the intervention.* Dose and engagement will be measured by the number of replies to messages received from participants and participant self-reported usage of and engagement with different components of the intervention (reported in the post-intervention survey). A subsample of participants in the intervention group will also be invited to participate in qualitative telephone interviews (with a researcher other than the interventionist). Qualitative interviews will gain more insight into what components of the intervention parents found useful and what they liked or disliked about components of the program

#### Secondary outcomes

##### Children’s objectively assessed sitting time

Children will wear an *activ*PAL™ for seven consecutive days pre and post intervention to objectively measure sitting time. The *activ*PAL™ has been shown to be valid, reliable and feasible in young children [33]. The *activ*PAL™ will be worn in the middle of the anterior aspect of the right thigh; the monitors will be sewn into purpose-made pouches affixed to leggings/bike shorts with Velcro, to be worn underneath normal clothes. Data will be collected in 15-s epochs and non-wear time will be defined as 10 min of consecutive zero counts and removed from daily wear time [34]. Participants will be required to have at least 6 h of wear time on at least 4 days, including one weekend day [34]. Where possible, participants will be asked to re-wear the *activ*PAL™ if they have insufficient data.

##### Parent-reported sedentary behaviour and screen time

Parents will report their child’s usual time in the last week in a range of sedentary behaviours including sitting down for reading/quiet play/craft activities and situations that restrict movement (e.g. in a car seat or stroller). They will also be asked to report their child’s usual time engaging in a range of screen-based behaviours (i.e. television viewing, computer use, electronic game use, smartphone and tablet computer use). Responses will be open (i.e. h/day and/or min/day) and the majority of items have previously established reliability [35].

##### Parent behaviours, knowledge and self-efficacy

Parents will be asked to report their own frequency and duration in physical activity in the previous week using the Active Australia Survey [36] and their usual week and weekend day television viewing [37]. Parents will also report their co-participation in physical activity and sedentary behaviour with their child, knowledge around physical activity and sedentary behaviour in early childhood, self-efficacy for promoting physical activity and limiting sedentary behaviour for their child, and an audit checklist of the home physical activity and sedentary behaviour environment [35, 38].

##### Sample demographics

Parents will be asked to report their own, their partner’s (if applicable) and their child’s demographic information (e.g. date of birth, parent education, parent employment status). Children’s height and weight will be measured at baseline by trained researchers using a Wedderburn portable rigid stadiometer, Wedderburn Tanita portable digital scales, and standardised measurement procedures [39, 40]. BMI will be calculated by standard formula (weight in kilograms divided by height in meters squared); BMI categories (healthy weight, overweight, obese) will be determined using age- and sex-specific international cut-off points [41].

### Statistical analysis

Analyses will be conducted using Stata 14 (StataCorp, College Station, TX, USA). Descriptive statistics will be used to describe the baseline characteristics of the sample. Feasibility and acceptability will be assessed using percentages and by analysing qualitative data, as appropriate. Linear and logistic regression will be used to determine the effect of the intervention on the secondary outcomes, controlling for potential confounders (e.g. child sex, age, BMI), baseline values and clustering by playgroup. Given the small sample size, effect sizes (Cohen’s *d* and Hedges’ *g*) will be calculated.

## Discussion

This paper presents the protocol for a pilot RCT to determine the feasibility and efficacy of a parent-focused, predominantly mobile phone-delivered intervention to reduce sedentary behaviour in 2- to 4-year-old children. Existing interventions to reduce sedentary behaviour in early childhood are scarce and report mixed results; few have been conducted with parents outside the preschool setting and many have limited potential for scalability [18]. Mobile phones have been rapidly adopted across most demographic groups [20], and offer a wide-reaching, low-cost channel for the delivery of health behaviour programs. However, they have not been extensively used in health behaviour programs for parents of young children [23]. Hence, small-scale RCTs are required to determine whether interventions delivered in this way are acceptable, feasible and practical for both participants and researchers [42].

Strengths of the current pilot study include the use of an objective measure of children’s sitting time and the large range of specific sedentary behaviours assessed (encompassing screen time, time spent restrained). In addition, the use of mobile phone technology to deliver the majority of the intervention content affords the potential for the intervention to be scaled-up and widely disseminated.

The findings of this study will be used to inform the development of larger-scale, mobile technology RCTs to support parents to minimise the amount of time their children spend in sedentary behaviour. Moreover, findings will contribute to the limited medical literature on interventions designed to support health behaviour during early childhood.

### Trial status

The trial commenced recruitment in June 2016. There are 59 participants enrolled, with the trial due to be completed in March 2017.

## Additional file

Additional file 1: SPIRIT Checklist. (DOCX 48 kb)

## Abbreviations

BMI: Body Mass Index
CONSORT: Consolidated Standards of Research Trials
RCT: Randomised controlled trial
SMS: Short message services
SPIRIT: Standard Protocol Items: Recommendations for Interventional Trials
